# Correction to: NASA Formal Methods

**DOI:** 10.1007/978-3-030-55754-6_26

**Published:** 2020-08-10

**Authors:** Ritchie Lee, Susmit Jha, Anastasia Mavridou, Dimitra Giannakopoulou

**Affiliations:** 8 NASA Ames Research Center, Moffett Field, CA USA; 9 SRI International, Menlo Park, CA USA; 10 KBR Inc., NASA Ames Research Center, Moffett Field, CA USA; 11 NASA Ames Research Center, Moffett Field, CA USA; 12 NASA Ames Research Center, Moffett Field, CA USA; 13 SRI International, Menlo Park, CA USA; 14 KBR Inc., NASA Ames Research Center, Moffett Field, CA USA; 15 NASA Ames Research Center, Moffett Field, USA

## Correction to: R. Lee et al. (Eds.): *NASA Formal Methods*, LNCS 12229, 10.1007/978-3-030-55754-6

The original versions of this book and Chapter 14 were revised. The following was corrected:

Dimitra Giannakopoulou, the General Chair of the NFM 2020 conference, was inadvertently forgotten and, therefore, added as a volume editor.

Chapter 14 was retrospectively made available open access under a CC BY 4.0 license at link.springer.com.

